# The Impact of Spine Injuries on Amateur Athletes: An Exploratory Analysis of Sport-Related Patient-Reported Outcomes

**DOI:** 10.3390/sports12080213

**Published:** 2024-08-01

**Authors:** Philipp Raisch, Tabea Hirth, Michael Kreinest, Sven Y. Vetter, Paul A. Grützner, Matthias K. Jung

**Affiliations:** Department of Trauma and Orthopedic Surgery, BG Klinik Ludwigshafen, University of Heidelberg, Ludwig-Guttmann-Straße 13, 67071 Ludwigshafen am Rhein, Germanypaul.gruetzner@bgu-ludwigshafen.de (P.A.G.)

**Keywords:** spine injury, spine trauma, return to sport, amateur athletes, patient-reported outcome

## Abstract

Introduction: There is a lack of information on return to sport and patient-reported outcome measures (PROMs) in amateur athletes after isolated spine injuries. Methods: A single-center cohort study in amateur athletes aged 18 to 60 with isolated spine injuries; clinical data collection and follow-up via telephone interview and standardized PROMs (Short-Form 36, Oswestry and Neck Disability Index, Tampa Scale of Kinesiophobia, Hospital Anxiety and Depression Scale, Pain Visual Analog Scale). Bivariate analyses of potential influencing factors on PROMs were conducted using the Wilcoxon Signed-Rank Test. *p*-values < 0.05 were considered statistically significant. Results: Out of the 80 included participants, 78% (n = 62) were active in sport at follow-up. PROMs were slightly worse than those described for the age-adjusted general population. There were consistent associations of better PROMs with having reached the subjective preinjury level of performance in sport, while injury severity and surgical or conservative therapy did not show consistent associations with PROMs. Conclusion: Most amateur athletes resume their sports activity after a spine injury. Better outcomes are associated with individuals’ resumption of sport and subjective level of performance, while injury severity and surgical or conservative therapy do not show consistent associations with PROMs, highlighting the importance of patient education, rehabilitation, and encouragement.

## 1. Introduction

### 1.1. Background

Traumatic spine injuries are rare [1], yet relevant, owing to the substantial impact they can have on an individual’s quality of life and physical functions [2]. Sufficient mobility and stability of the spine are paramount, especially during sports, as athletes in different sports transfer high levels of force through the spine [3]. As a result, problems concerning return to sport (RTS) can occur after a spine injury.

Additionally, psychological sequelae after trauma, like anxiety or fear of movement and reinjury, might hinder patients from returning to sport. Patients with fear of movement and reinjury present higher disability levels, independent of biomechanical status and pain intensity [4]. Possible problems extend beyond the field of recreational sports, as there is an association between sports activity and health-related quality of life [5].

To date, the return to sport after various pathological conditions of the spine has been investigated mainly in the context of professional sports. Most professional athletes are able to return to sport after surgery for different conditions of the cervical spine [6], including cervical spine injuries [7]. After injuries and other conditions of the lumbar spine, most professional athletes not only safely return to sport [8,9] but also reach their preinjury level of performance [8]. These findings are exemplary for a great body of scientific literature dealing with professional athletes’ RTS after spine injury or degenerative conditions.

This is contrasted by a relative paucity of information on RTS and sport-related patient-reported outcomes (PRO) in amateur athletes. There are reports of amateur athletes returning to sport after elective surgery for degenerative conditions of the cervical spine [10,11]. In a literature review, including athletes of any level, Alsobrook et al. concluded that athletes with conservative or minimally invasive treatment for lumbar spinal disorders have the greatest likelihood of RTS and reaching their preinjury level of performance, while those with more severe conditions, such as compression fractures, and more invasive procedures like spinal fusions, were less likely to return to sport and were discouraged from returning to collision sports [12].

In summary, available studies allow only limited conclusions on RTS in amateur athletes after spine injury, not only because they mainly focused on professional athletes but also because they included patients treated for different conditions of the spine, not focusing on traumatic injury. On the other hand, studies describing outcomes and functional recovery after spine injury specifically, omitting patients treated for degenerative conditions, included patients with concomitant injuries like spinal cord injury, extremity fractures, or structural brain injury [13]. This makes it hard to discern the impact of isolated spine injuries. And while it is clear that neurological status is the most important predictor of functional outcomes after a spine injury [14], there is a lack of information on other influencing factors investigated in patient samples without spinal cord injury, such as age, comorbidities, injury severity, and patients’ preinjury engagement in sport. Additionally, only a small proportion of available studies employ patient-reported outcome measures (PROMs) [8,10].

As a result, evidence-based guidelines on RTS after a spine injury for amateur athletes are missing [15] and patients and treatment teams are often unsure about the expected functional recovery and prognosis regarding sports activity after a spine injury.

### 1.2. Aims

The aim of this study was to describe PRO related to sport after isolated injuries to the spine in amateur athletes. Changes in sports activity, subjective level of performance, and sports disciplines should be characterized. In light of a growing focus on PROMs in clinical spine research [16], we aimed to describe various aspects of PRO. Here, we wanted to explore the connections of well-characterized PROMs in patients with a spine injury to RTS status, as well as previously less well-studied factors, such as age, sex, comorbidities, injury morphology, as well as treatment. The choice of PROMs should be based on the above-characterized connections between spine injury, quality of life, fear of movement and reinjury, and disability [2,4,5], in order to ultimately contribute to more reliable prognoses for amateur athletes sustaining spine injuries.

## 2. Materials and Methods

This cohort study was conducted at a national trauma center in Germany. It was approved by the local ethics committee (Ethics Committee of the State Medical Association Rhineland-Palatinate, Mainz, Germany; application number 2022-16283). Patient data were analyzed retrospectively, and mid-term follow-up was acquired via telephone interviews and standardized questionnaires. Reporting of this study is based on the STROBE Guidelines [17].

### 2.1. Participant Selection and Inclusion

Patients treated at the study clinic from 2016 to 2020 because of structural injuries of the spine (fractures or discoligamentous disruptions) were retrospectively screened for inclusion and exclusion criteria (Table 1, category A). This window of time was chosen to ensure that a minimum of 1.5 years since the injury had passed at the time of the data collection in 2022, while not including patients treated before 2016, in order to minimize recall bias. To minimize the risk of including patients with osteoporotic fractures, patients were included if they were not over 60 years old at the time of the injury and a traumatic event was clearly defined. They were excluded if they had a pathological spine fracture or had suffered their injury in an attempted suicide. Patients were also excluded if they had relevant concomitant injuries like fractures of the pelvis, the extremities, or structural brain injuries.

Potentially eligible patients were contacted in reversed chronological order, starting with the individuals treated most recently. A telephone interview specifically designed for this study, was conducted, and further criteria for inclusion or exclusion were determined (Table 1, category B). Here, patients were not contacted if they were under 18 years of age. They were not included in the study if they had not been active in sports before the injury, had insufficient language comprehension, or did not consent.

After the interview, standardized questionnaires were sent to participants. Participants who did not send back their questionnaires after being reminded or only filled them out incompletely were excluded from the analysis.

### 2.2. Injury Treatment and Posttreatment Recommendations

The decision on surgical or conservative treatment was based on national guidelines [18,19,20], considering patient-specific factors and informed consent. As the standard of care at the study site, all patients received early functional therapy directly after surgery. For further outpatient treatment, the continuation of physical therapy was recommended. Patients were advised against carrying weights greater than ten kilograms for six weeks and against manipulations of the spine. Full weight bearing was allowed after six weeks after injury or surgery. It was recommended patients not partake in activities with high axial loads or abrupt changes in direction for six months after surgery or injury.

### 2.3. Data Collection

Data on demographic characteristics, concomitant injuries, and preexisting conditions were collected retrospectively from the hospital database and were complemented with information later acquired via telephone interviews. The Charlson Comorbidity Index (CCI) was calculated [21].

Spine injuries were classified according to the applicable AO Spine Classification [22,23,24] based on post-injury CT scans. Type A injuries are bony injuries only without significant ligamentous or disc injuries. In most instances, A0, A1, and A2 fractures are considered stable, allowing for conservative therapy, while A3 and A4 injuries describe fractures with increasing involvement of the vertebral body, often necessitating surgical stabilization. Type B injuries involve the tension bands and/or discs and are unstable, necessitating surgery in most cases, while type C injuries are highly unstable translational injuries, necessitating surgery and carrying a high risk of spinal cord injury. In the cervical spine, special classifications for facet joint injuries are present (F1–F4), depending on the area of the facet joints involved, as well as facet dislocation.

Treatment data, surgical and conservative, were extracted along with information on revision surgery or implant removal, which was also complemented with information from the telephone interview.

### 2.4. Telephone Interview

A standardized telephone interview in German, specifically designed for this study, was developed in 2021 by the authors (English translation in Supplementary Materials). In the first part, general information on posttreatment and rehabilitation and preexisting medical or spinal conditions (e.g., scoliosis, chronic back or neck pain, disc protrusions) were queried, as well as whether any revision surgery had been performed elsewhere. In the second part, information on sports activity before and after the injury was collected, including disciplines of sport, time spent on sport per week, any problems during sport, and whether participants had subjectively reached their preinjury level of performance (subjective preinjury level of performance, sPILP) after the injury. Since only qualitative information on treatment and preexisting conditions and no psychometrical information was deduced from this interview, no prior validation of the interview was done.

As “amateur sport”, we defined any regular physical activity performed in participants’ free time not involving remuneration, and as “amateur athlete”, any person participating in such activities. “Return to sport” was defined as participants returning to “amateur sport” irrespective of performance level or competition.

### 2.5. Patient-Reported Outcome Measures

The German versions of the following standardized and well-validated questionnaires were sent to participants. The Short-Form-Health-Survey-36 [25], from which the Physical Component Score (SF-36 PCS), from 0 to 100, was calculated to measure the health, possible pain, and health perception of participants. Participants with injuries of the thoracolumbar spine were sent the Oswestry Disability Index (ODI) [26], and participants with injuries of the cervical spine, the Neck Disability Index (NDI) [27]. Participants with injuries to both regions received both questionnaires. The ODI and NDI measure back-pain- and neck-pain-specific disability on a scale from 0 (no disability) to 100 (maximum disability).

The 17-item version of the Tampa Scale of Kinesiophobia (TSK-17) measures increasing fear of physical activity and reinjury on a scale from 17 to 68, with 37 points being the cut-off for kinesiophobia [28]. The Hospital Anxiety and Depression Scale (HADS) measures symptoms of anxiety and depression, with points from 0 to 42; 15–21 points marking moderate and >21 points, severe stress [29]. Participants were also sent visual analog scales to rate their maximum neck or back pain during the last five days from 0 to 100 as well as, based on an approach by Langenfeld et al. [30], their cervical or thoracolumbar subjective ROM in the directions flexion/extension, lateral bending left/right, and rotation left/right. To the authors’ knowledge, this original score has only been used once before, and the derived score for the thoracolumbar spine has not yet been validated.

### 2.6. Statistical Analysis

The above-mentioned baseline variables and PROMs were entered into a database which was analyzed using IBM SPSS Statistics Ver. 27 (IBM, Armonk, NY, USA). Data on demographics, preexisting conditions, treatment, sports activity, subjective ROM, and any problems during sport after the injury were reported descriptively.

In addition to descriptive reporting, PROMs were also compared among various participant subgroups, divided according to different patient-, injury- and treatment-related characteristics, using the Wilcoxon Signed-Rank Test. *p*-values < 0.05 were considered statistically significant. There was no adjustment for multiple testing in this exploratory study.

## 3. Results

### 3.1. Participant Recruitment

A total of 94 patients were contacted and agreed to partake in the study. After the structured telephone interview, 80 participants (85%) sent back complete questionnaires and were included in this analysis.

### 3.2. Demographics and Preexisting Conditions

Participants’ mean age at the time of the injury was 45.1 years (SD 13.5, range 16 to 60); 38% were female (n = 30); and 62% male were (n = 50, Table 2). The mean time from injury to the interview was 3.2 years (SD 1.1, range 1.5 to 5.6). The burden of preexisting medical conditions was low, with only 18% of participants having a CCI greater than 0 (n = 14) and a median CCI among all participants of 0 (IQR 0, range 0 to 4). However, most participants, 58% (n = 46), reported having preexisting pathologies of the spine. Mean BMI was 25.6 kg/m^2^ (SD 4.8, range 17.9 to 46.9), and 46% of participants (n = 37) were formally overweight with a BMI > 25 kg/m^2^.

### 3.3. Injury Morphology and Treatment

An isolated cervical spine injury was present in 18% of participants (n = 14); only the thoracic spine was injured in 28% (n = 22), and only the lumbar spine in 40% (n = 32), while 15% of participants had injuries to multiple regions of the spine (n = 15, Table 3). As per inclusion criteria, no participant had a spinal cord injury. The most frequent injury morphologies according to the appropriate AO Spine Classification were A3 in 35% (n = 28) and A4 in 21% (n = 17), followed by A1 in 11% of participants (n = 9, Table 3). In total, surgery was performed on 69% of participants (n = 55), and 31% were treated conservatively (n = 25). Postoperatively, there were three cases of a wound-healing disorder in the thoracolumbar spine requiring revision surgery (5% of surgical participants). No other revision surgeries elsewhere were reported in the telephone interview.

The injury had been sustained in a sports accident in 24% of participants (n = 19). In the telephone interview, 84% reported having received physical therapy after discharge (n = 67), while 50% also reported having received rehabilitation (n = 40).

### 3.4. Sports Activity before and after the Injury

**Figure 1:**
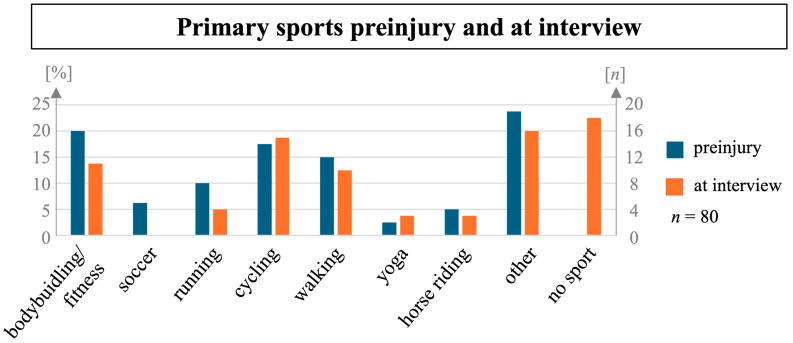
Relative and absolute frequency of different primary sports preinjury and at the time of the interview.

The most frequent primary sports before the injury were bodybuilding/fitness (20%, n = 16), cycling (18%, n = 14), and walking (15%, n = 12, Figure 1). Of the participants, 45% reported having spent at least five hours a week on sport before the injury (n = 36), and 56% reported having been active in sports at least three times a week (n = 45).

At the time of the interview, 78% of participants reported being active in sport (n = 62), while 22% were not active in sport (n = 18). Here, the most frequent primary sports were cycling (19%, n = 15), bodybuilding/fitness (14%, n = 11), and walking (13%, n = 10, Figure 1). Comparing only participants active in sport before and after the injury, there was a decrease in the popularity of bodybuilding/fitness, soccer, running, and horse riding after the injury and an increase in participants reporting cycling, walking, and yoga as their primary sport. At the time of the interview, 55% of participants active in sport reported having reached their subjective preinjury level of performance (sPILP, n = 34).

### 3.5. Reported Problems during Sport 

**Figure 2:**
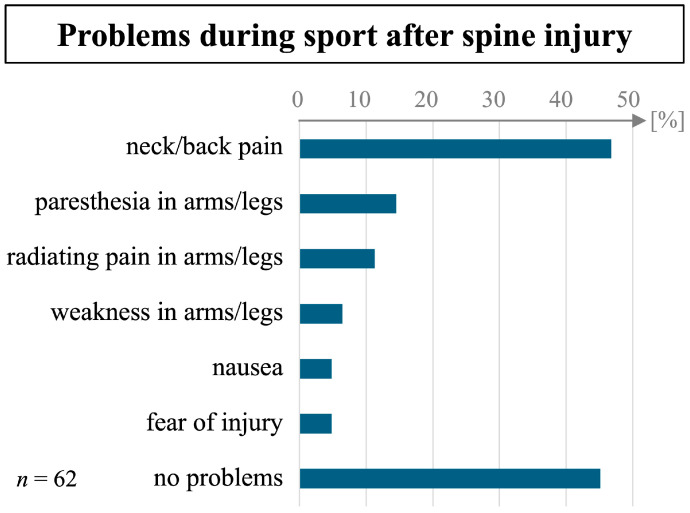
Relative frequency of reported problems during sport in participants who returned to sport after a spine injury.

The 62 participants, who were active in sport at the time of the interview, were asked about current problems they experienced during sport (Figure 2). Only 45% (n = 28) reported no problems at all, while the most frequent problem was neck and/or back pain in 47% (n = 29). Much less frequent were paresthesia (15%, n = 9) or weakness in arms or legs (6%, n = 4), as well as nausea (5%, n = 3). Fear of injury during sport was reported by 5% (n = 3).

### 3.6. Subjective Range of Motion

Participants were asked about their subjective ROM in the regions of the spine affected by injury on an analog scale from 0 to 100 for different directions of movement (Table 4). While most participants reported some subjective restriction of ROM, median values for different directions both in the cervical as well as the thoracolumbar spine were above 70%, with the exceptions of lateral bending to the left in the cervical spine (median 64%) and extension in the thoracolumbar spine (median 53.5%). Perceived ROM tended to be reported higher in the cervical spine.

### 3.7. Standardized Patient-Reported Outcome Measures and Potential Influencing Factors

**Figure 3:**
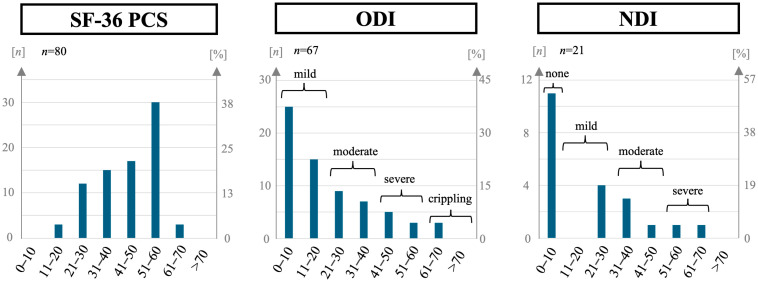
Patient-reported outcome scores for health-related quality of life (Short-Form-36 Physical Component Score, SF-36 PCS, n = 80), back-pain-specific disability (Oswestry Disability Index, ODI, n = 67), and neck-pain-specific disability (Neck Disability Index, NDI, n = 21) in amateur athletes after a spine injury.

**Figure 4:**
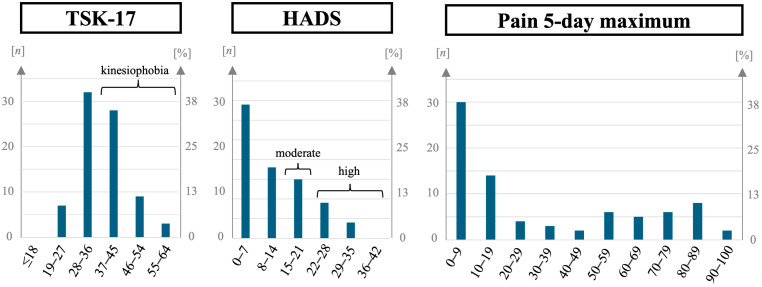
Patient-reported outcome scores for fear of movement and reinjury (Tampa Scale of Kinesiophibia 17 Item Version, TSK-17), symptoms of anxiety and depression (Hospital Anxiety and Depression Scale, HADS), and maximum pain in neck or back over the last five days (visual analog scale from 0 to 100) in amateur athletes after spine injury. n = 80.

Participants received standardized PROMs (Table 5, Figure 3 and Figure 4). To explore potential influences on PROMs, we performed bivariate analyses in various subgroups divided by baseline variables concerning sports activity, preexisting conditions, injury, treatment, posttreatment, and motivation using the Wilcoxon Signed-Rank Test (Table 6 and Table 7). Concerning the time passed between the injury and the interview, there were no significant differences between the group with above- and the group with below-average time of 3.2 years, suggesting a negligible effect of further time passing after the minimum time of 1.5 years among our participants on the investigated PROMs.

The mean of the PCS of the SF-36 was 44.5 points out of 100 (95% CI) for the mean [41.7;47.1], median of 46 points, range 16 to 68, Table 5, Figure 3). Here, significantly better scores were observed in participants with RTS at the time of the interview (*p* = 0.044, Table 6), and, among those with RTS, in participants who reported having reached their sPILP (median 55.5 vs. 38.5 points, *p* < 0.001). The subgroups who were younger than average (*p* = 0.005), had a CCI of 0 (*p* = 0.016), or had no preexisting spine conditions (*p* = 0.007), also scored significantly better. On the other hand, treatment- or injury-specific subgroups did not differ significantly (Table 6).

The mean ODI (Table 5, Figure 3), measuring back-pain-specific disability, was 19.6% (95% CI [15.2;24.0], median 12%, n = 67, range 0 to 68%). There was no significant difference based on RTS status (*p* = 0.082). However, among those with RTS, those reporting having reached their sPILP scored significantly better (median 8.0% vs. 26.0%, *p* = 0.001, Table 6). Significantly worse ODI values were seen in participants of higher age (*p* = 0.036) and with preexisting spine pathology (*p* = 0.001) while having multiple regions of the spine affected was the only treatment- and injury-related subgroup characteristic that had statistical significance (*p* = 0.036). NDI, measuring neck pain-specific disability (Table 5, Figure 3), was available for n = 21 participants with cervical spine injury with a mean of 18.0% (95% CI [9.4;26.6], median 18%, range 0 to 33%), showing notably better results for the subgroup who stated having reached their sPILP (median 3.0% vs. 28.0%, *p* = 0.002, Table 6).

The mean for the TSK-17 (Table 5), measuring fear of movement and reinjury, was 36.9 out of 68 (95% CI [35.1;38.8], median 37 points, range 21 to 60). Forty-five percent (n = 36) had a score over 37 points, indicating kinesiophobia (Figure 4). Significantly worse results were present for participants without RTS (*p* = 0.016), without having reached their sPILP (*p* = 0.002), and with preexisting spine pathology (*p* = 0.037), as well as for participants who had not sustained their injury during sport (*p* = 0.005), meaning that participants with a sports injury were less afraid of reinjury than those who had sustained their injury elsewhere (Table 7).

Mean HADS (Table 5), measuring symptoms of anxiety and depression, was 11.7 out of 42 (95% CI [9.9;13.6], median 9 points, range 0 to 34). Scores indicating moderate stress (15–21 points) were present in 19% (n = 15), and scores indicating severe stress (>21 points) in 16% (n = 13, Figure 4). Again, results were significantly worse in participants who had not reached their sPILP (*p* < 0.001). Considering the injury, a singular cervical spine injury resulted in significantly better (*p* = 0.017) and having multiple regions of the spine affected in worse results (*p* = 0.018), while treatment and patient characteristics showed no significant association (Table 7).

Maximum pain in the five days before the interview (Table 5, Figure 4) had a mean of 31.2 on a visual scale from 0 to 100 (95% CI [24.1;38.3], median 16.5, range 0 to 94). Both RTS (*p* = 0.025) and having reached the sPILP (*p* < 0.001) were associated with lower pain scores (Table 7). Notably, in the latter, median pain scores were 4.5 in individuals who had and 51.5 in individuals who had not reached their sPILP. Participants with a CCI greater than 0 also had significantly higher pain scores (*p* = 0.015), while an isolated cervical spine injury was associated with less pain (*p* = 0.033).

Looking at posttreatment, there was a consistent association between the need for rehabilitation and worse outcomes for all PROMs (Table 6 and Table 7), with significantly worse scores for the SF-36 PCS (*p* < 0.001), ODI and NDI (*p* = 0.001 and *p* = 0.023), TSK-17 (*p* = 0.002), HADS (*p* < 0.001), and pain (*p* < 0.001). The need for physical therapy was associated with worse ODI scores (*p* = 0.019) and higher pain scores (*p* = 0.005).

Finally, whether participants were more or less active in sport before the injury did not significantly influence any PROM, with the exception of the HADS, which scored slightly higher in the subgroup with at least three times of sport a week (median 12 vs. 7, *p* = 0.031, Table 7).

## 4. Discussion

In this cohort study of 80 amateur athletes who had sustained an isolated spine injury, we described RTS, changes in preferred disciplines, and patient-reported problems during sport, as well as standardized PROMs. The latter were then analyzed for potential influencing factors. At the time of the interview, at an average of 3.2 years after the injury, 78% of participants had resumed sport, and 55% of those with RTS reported having reached their sPILP. There was a shift in primary sports toward low-impact sports such as cycling and yoga, and 55% of participants still reported various problems during sport, the most frequent being pain, reported by 47%. Restrictions in subjective ROM of the cervical and thoracolumbar spine were also prevalent. Standardized PROMs on health-related quality of life, back- and neck-pain-specific disability, fear of movement and reinjury, as well as anxiety and depression, scored slightly worse than the general German population. Pain was a frequent problem. There were consistent associations between better outcomes with having reached the sPILP in sport and associations with worse outcomes with the need for rehabilitation, while injury severity and surgical or conservative therapy did not show consistent associations with PROMs.

The scientific literature on RTS in amateur athletes after spine injury is limited. In contrast, RTS in professional athletes has been diligently studied, showing that RTS rates and outcomes for most isolated spine conditions, including trauma, are generally favorable, and most professional athletes can return to sport at their preinjury level of performance [6,7,9,15].

However, results from these studies can hardly be extrapolated to amateur athletes since great differences in preinjury fitness, motivations for returning to sport as well as resources available for rehabilitation, are to be expected.

In a prospective series of 50 cases, Reinke et al. investigated RTS after elective cervical disc replacement between 2006 and 2012, including 24 amateur athletes ages 18 to 60 [10]. All amateur athletes returned to sport after surgery. PROMs before and after surgery were queried using a modified Tegner score, showing no significant differences before and after surgery for the whole cohort. However, these results must be compared to ours with caution, as patients undergoing elective surgery are expected to suffer some disability concerning their spine before the surgery and to improve over time after surgery compared to the baseline.

The same limitations apply to a cohort study by Richards et al. from 2020 [11], who asked a cohort, including 53 amateur athletes, about their RTS after elective surgery of their cervical spine, determining an RTS rate of 81.1% (n = 43). In addition to the fact that only the disciplines swimming, golf, and tennis were investigated, comparisons to our findings are further hampered by the fact that the cohort’s median age was 69 years, ranging from 33 to 90 and, thus, much higher than in our study.

Knop et al. conducted a prospective multicenter study on the results of surgery for thoracolumbar spine injuries in 2001 [13]. Out of 372 patients with follow-up, only 48% returned to their preinjury physical activity level. While no details on specific activities such as sports disciplines were reported, these much worse outcomes can be explained at least in part by the fact that 71 patients with spinal cord injury, as well as patients with multiple traumas, were included. This underlines the importance of studying patients with isolated spine injuries if one is to discern the effect these particular injuries have on physical functioning.

A systematic literature review by Alsobrook et al. from 2008 summarized studies on RTS in athletes after various conditions of this anatomic region of the spine [12]. In this review, no clear distinction between professional and amateur athletes was made, so it is unclear to what degree their findings can be applied to the general population participating in amateur sports. Concerning fractures of the lumbar spine, they concluded that spinal process fractures could usually be treated conservatively, and RTS, even to collision sports, was generally possible. However, athletes requiring more invasive therapy, like surgical stabilization, for any condition were less likely to return to sport at their preinjury level of performance, and collision sports were generally discouraged. Contrary to this, we found no consistent association between PROMs and injury severity or surgical vs. conservative therapy in our cohort. This emphasizes the observation made in most literature reviews that evidence levels concerning RTS recommendations after spine injury are still low owing to a paucity of high-quality clinical studies [6,9,15].

### 4.1. Changes in Sport Disciplines

In our cohort, 78% of participants reported being active in sport at the time of the interview. There are no evidence-based recommendations for ideal sport after spine injuries [15], but sports leading to high levels of repetitive forces being transmitted through the spine [3] might naturally be avoided by individuals with spine injuries. Accordingly, comparing sports before and after the injury in our cohort, we saw an absolute and relative increase in low-impact sports like yoga and cycling as primary sports, while bodybuilding/fitness, soccer, running, walking, and horse riding, generally associated with higher strain on the spine, all saw decreases in popularity. A decline in the popularity of high-impact sports like running, skiing, and climbing was also seen in the cohort of patients with elective cervical spine surgery by Reinke et al. [10]. Accordingly, Richards et al. saw much lower RTS rates for tennis (31.2%) than for golf (67.6%) and swimming (81.6%) [11]. Following this observation, it is desirable to offer patients evidence-based guidelines on which sports are best suited to continue physical activity after spine injuries. It is also possible that patients’ families or therapists might have influenced the choice of sports discipline after injury. However, data are not able to answer these questions.

### 4.2. Reported Problems during Sport and Range of Motion

The high percentage of participants still reporting problems during sport at a minimum of 1.5 and a mean of 3.2 years after the injury underlines the importance of research into amateurs’ RTS after a spine injury. Less than half the participants in our cohort (45%) with RTS reported having no problems at all during sport, with pain being the most frequent problem (47%). Participants also reported differing degrees of subjective ROM restriction. It is interesting in this context that many guidelines recommend RTS only once athletes are pain-free, at a full range of motion, and at full strength [15], while amateur athletes seem to return to sport despite persisting problems regarding their spine injuries. The subjective ROM score used here is a novel tool based on an approach by Langenfeld et al. [30]. It was adapted for the thoracolumbar spine for this study. Since there is no broad application and validation of this measurement tool, results should be interpreted cautiously.

### 4.3. Patient-Reported Outcome Measures and Influencing Factors

Health-related quality of life: Mean SF-36 PCS in our cohort was 44.5 out of 100 (95% CI [41.7;47.1]), which lies lower than the values of 50.4 to 55.8 reported for different age groups between 18 and 60 years in the German population [31]. This highlights the impact of even isolated spine injuries without spinal cord injury on patients’ health-related quality of life. Better PCS values were associated with RTS and having reached the sPILP, which underlines the role of physical activity for health-related quality of life [5] after a spine injury. However, a lower PCS in our cohort was also associated with higher age and accordingly, with preexisting medical and spinal conditions, as seen in the general population [31]. It is interesting to note that injury- and treatment-specific variables like fracture severity, surgical or conservative treatment, or having multiple regions of the spine affected did not result in significant differences.

Back- and neck-specific disability: For ODI and NDI, which were queried based on injured regions of the spine, our cohort scored means of 19.6% and 18.8%, respectively (95% CI [15.2;24.0] and [9.4;26.6]). This lies within the ranges of “mild” to “moderate” disability for the ODI [26] and “mild” disability for the NDI [32]. Notably, there was no significant association between these popular outcome tools and RTS status, only with the sPILP. While the ODI was significantly higher in surgically treated patients (medians 20% vs. 10%, *p* = 0.001), the disability based on these values is still considered “mild” even for surgically treated patients [26]. The observed association of higher age and preexisting conditions of the spine with worse ODI is to be expected [33].

Fear of movement and reinjury: Fear of movement and reinjury was assessed using the TSK-17. With 37 points representing the described cut-off for kinesiophobia [28], 45% of our cohort (n = 36) showed signs of increased fear of movement and reinjury. This contrasts with answers in the telephone interview, where only three participants reported fear of injury during sport. While statistically significant associations of kinesiophobia to RTS and sPILP were present, the absolute differences between medians were only moderate. It is plausible, however, that participants with a higher fear of movement might hesitate to take up sport after injury. Reciprocally, the literature also supports that exercise can reduce fear of movement in patients with low back pain [34].

Clinically and statistically significant subgroup differences in kinesiophobia for patient, injury, or treatment characteristics were absent. An exception was markedly lower values for kinesiophobia in participants who had sustained their injury in a sports accident (medians 30.0 vs. 38.5, *p* = 0.005). While at first contrary to intuition, this might be due to an association between a lower fear of injury before the spine injury and an affinity to higher-risk sports, resulting in injury. If so, then these participants seem to have preserved their low fear of injury even after a sports accident had occurred.

Anxiety and depression: Mean HADS, measuring symptoms of anxiety and depression, was 11.7 points out of 42 (95% CI [9.9;13.6]). This is comparable with values in the corresponding general German population up to the age of 60 years, with means reported from 6.8 to 10.4, depending on gender and age [29]. However, with 35% of participants in our cohort above the threshold of 15 for moderate stress, this proportion was greater than in comparable cohorts in the general German population, where percentages of 13.3% to 24.4%, depending on gender and age, are reported [29]. While an increased risk for mental health issues in individuals with spinal cord injury is known [35], we found no such association for individuals with isolated spine injury described in the literature. This issue therefore warrants further research.

Pain: Finally, the maximum pain over the past five days was queried on a visual analog scale from 0 to 100, with the mean pain score being 32.2 (95% CI [24.1;38.3]) and a median of 16.5. Significant associations of higher pain with missing RTS and not having reached the sPILP are plausible. Interestingly, medical comorbidities were also significantly associated with higher pain values, while preexisting spine pathologies were not. Neither did injury severity nor therapy modality show significant associations.

There was no association of overweight (BMI > 25 kg/m^2^) with any PROM. Indeed, there are data showing that obese patients undergoing spinal fusion surgery have a higher risk for adverse events [36], can profit equally and show similar improvement in outcome parameters [36,37] compared to non-obese patients. According to our data, preinjury motivation, measured by frequency and duration of sport before the injury, did not play a significant role in PRO. However, in the literature, there are reports on an association between lack of training and unfavorable functional outcomes after fractures of the thoracic and the lumbar spine [13].

Of special note, no significant differences were seen for any of the PROMs dependent on injury severity or surgical vs. conservative therapy, with the exception of the ODI, as stated above. One might, therefore, speculate that medical advances and modern treatment guidelines have come a long way toward offering optimal surgical or conservative care based on injury- and patient-specific parameters [18,19,20], at least for this patient group with a relatively young age and preinjury physical activity history. This also highlights the importance of patient education, rehabilitation, and encouragement, as having a substantial influence on the process of healing and returning to activity after spine injury and need to be present after the acute surgical or conservative treatment phase.

Interestingly, out of all the analyzed potential influencing variables, rehabilitation after the injury was most consistently associated with worse outcomes in all investigated measures. This is most likely due to the confounder of protracted healing after injury being associated both with the need and application of rehabilitation as well as worse PRO. The meaning and benefit of rehabilitation are recognized in a German S2k guideline after injury of thoracolumbar spine injuries [38], recommending early functional movement-oriented therapy, including sports therapy, and improved functional outcomes after thoracic and lumbar spine fractures have been shown after rehabilitation [13]. The role of rehabilitation in individuals with protracted healing after spine injury is, therefore, a relevant field of future research, as the question stands whether more intense or earlier rehabilitation in the affected patients might be able to achieve more favorable results.

### 4.4. Practical Implications and Future Research

In summary, while most amateur athletes in our cohort were able to return to sport after their spine injury, the proportion of individuals who did not is considerable (22%). Additionally, most participants did not return to their sPILP. Both not returning to sport, as well as not reaching one’s sPILP, were consistently associated with worse PROMs, demonstrating a need to optimize patient care. Interestingly, more severe injury morphology, as well as whether surgery was necessary or not, did not show consistent associations with PROMs. This suggests that the key to better RTS rates, performance levels, and PROMs does not lie in optimizing surgical decision-making or surgical treatment. This is further underlined by the fact that the need for rehabilitation was consistently associated with worse PROMs. One might deduce that improvements in post-acute care after discharge from the hospital, involving physical therapy, inpatient rehabilitation, and possibly patient education and encouragement, could lead to better RTS rates and PROMs.

Future research should investigate specific reasons for why individuals do not return to sport or reach their sPILP and what role the quality and quantity of their post-acute care might play in this. Also, the association of rehabilitation with worse outcomes must be further investigated, looking at the point in time after the injury rehabilitation takes place and whether earlier escalation of posttreatment to inpatient rehabilitation could improve results. Following this, indicators of patients in need of rehabilitation should be identified.

Even when focusing on the pathological category of trauma, the patient cohort can be heterogeneous, as seen in our study. Following this exploratory analysis, larger studies on sport-related PROMs after specific spine injuries with more homogenous patient samples are desirable to allow for the development of evidence-based recommendations on the optimal point in time and sports disciplines to return to after spine injuries.

### 4.5. Limitations

Important limitations of this study arise from the difficulty of extrapolating our results to a general population of patients with spine injuries. This is a single-center study, conducted at a national trauma center, and we only included individuals treated as in-patients. Therefore, a selection bias towards patients with more complex spine injuries and towards surgically treated patients is present, as some conservatively manageable cases might have been treated as outpatients, or no referral to our institution might have taken place. There is also selection bias towards individuals with adequate German language skills due to the inclusion criteria requiring the comprehension of the questionnaires.

A broad spectrum of patients was included, with injuries to different regions of the spine and different treatment modalities. Different sports activities before the injury were included, in sum, introducing significant heterogeneity. At the same time, due to our strict inclusion criteria concerning concomitant injuries and age, our sample size was limited to only 80 participants. As a result, no detailed analyses of injury- or treatment-related subgroups were performed.

The time between the injury and the interview had a mean of 3.2 years, ranging from 1.5 to 5.6 years. This introduces the problem of substantial differences in the time of healing and rehabilitation between participants. We tried to account for this by testing for significant group differences in outcome measures between those above and those below the average follow-up time of 3.2 years and did not detect any. However, the differing follow-up durations in our cohort introduce further heterogeneity.

Also, the definitions of “amateur sport” and “return to sport” were chosen liberally, including any regular physical activity participants performed in their free time that did not involve remuneration, irrespective of performance level or competition. This needs to be considered when comparing our results to studies that possibly employ a stricter definition.

As specified in the inclusion criteria, there is no information on important patient groups, such as those with spinal cord injury. We also did not include patients beyond the age of 60 to minimize the effects of comorbidities and osteoporotic fractures, which are an entity to study separately. However, it must not be neglected that sports activity is increasingly important for the older population [39].

Addressing statistical analyses, we did not adjust for multiple testing, as the aim of this exploratory study was to facilitate the formation of hypotheses regarding future research into amateur sports activity after a spine injury. Naturally, any significant results must be interpreted cautiously.

To our knowledge, this is the first systematic investigation into RTS in amateur athletes after a spine injury, with strict exclusion of concomitant injuries, painting a clearer picture of the influence of spine injuries on sports activity and sport-related PRO than has previously been available. The application of standardized PROMs should facilitate comparisons with further larger scale and, ideally, prospective investigations.

## 5. Conclusions

Spine injuries are an event of considerable impact for amateur athletes. While a great proportion resumes their sports activity, only a minority reaches their subjective preinjury level of performance. Persisting problems, especially pain and restriction in subjective range of motion, are frequent, possibly contributing to changes in sports activity toward lower-impact disciplines.

Comparisons to the general population regarding standardized patient-reported outcome measures on health-related quality of life, disability, fear of movement, anxiety, and depression, as well as pain, demonstrated the impact of spine injuries on patients’ well-being and functional status. Better outcome parameters were associated with individuals’ resumption of sport and, especially, their subjective level of performance, while worse outcomes were associated with the need for rehabilitation as a sign of protracted healing. Injury severity and surgical or conservative therapy did not show consistent associations with outcome measures. This suggests that after successful primary care, patient education, rehabilitation, and encouragement should be emphasized by treatment teams to facilitate patients’ RTS and improve PROMs.

Future research should investigate the role of posttreatment, especially rehabilitation, in order to identify patients in need of early escalation of posttreatment. A larger case series of more homogenous patient samples is needed, looking into the relationships among patient characteristics, injury morphology, sports activity, and PROMs to lay the groundwork for evidence-based guidelines on RTS after a spine injury.

## Figures and Tables

**Table 1:** Criteria for inclusion and exclusion.

**A.** **Criteria Determined in Retrospective Analysis**	
**Inclusion**	**Exclusion**
Inpatient treatment at the study site between 2016 and 2020	Pathological fracture (including osteoporotic fracture)
Age at injury ≤60 years	Injury caused by attempted suicide
Time of trauma clearly defined	Relevant concomitant injuries
Vertebral fracture or discoligamentous injury	- Spinal cord injury - Structural brain injury - Injuries of extremities necessitating conservative treatment >2 weeks or surgery - Pelvic fracture
**B.** **Criteria Determined via Telephone Interview and Questionnaires**	
**Inclusion**	**Exclusion**
Active in sports before injury	Insufficient language comprehension
Age at interview ≥18 years	Questionnaires incomplete
Informed consent	

**Table 2:** Sex, Age, preexisting medical and spinal conditions, and time from injury to interview.

Sex		
Women, n (%)	30	38%
Men, n (%)	50	63%
**Age at injury** (mean, SD, range)	45.1 years	(SD 13.5, 16–60)
**Preexisting conditions**		
Charlson Comorbidity Index (median, IQR, range)	0	(IQR 0, 0–4)
Body Mass Index (mean, SD, range)	25.6 kg/m^2^	(SD 4.8, 17.9–46.9)
Preexisting spine pathology, n (%)	46	58%
**Time from injury to interview** (mean, SD, range)	3.2 years	(SD 1.1, 1.5–5.6)

Abbreviations: SD, standard deviation; IQR, interquartile range.

**Table 3:** Injured regions of the spine, injury morphology according to AO Spine, and treatment.

	Distribution			Treatment	
		n	%	Conservative (n)	Surgical (n)
**Injured region**	Cervical	14	18	4	10
	Thoracic	22	28	10	12
	Lumbar	32	40	8	24
	Multiple	12	15	3	9
**Injury morphology** (AO Spine) *	A0	3	4	3	0
	A1	9	11	9	0
	A2	5	6	1	4
	A3	28	35	10	18
	A4	17	21	0	17
	B1	1	1	0	1
	B2	5	6	0	5
	B3	3	4	0	3
	C	7	9	0	7
	F1	1	1	1	0
	F2	1	1	1	0

* In case of multiple injured regions, the most severe injury is given.

**Table 4:** Participants’ subjective range of motion in different directions on a scale from 0 to 100 for different regions of the spine.

	Cervical Spine	n = 21	Thoracolumbar Spine	n = 66
	Median (IQR)	Min–Max	Median (IQR)	Min–Max
Flexion	83 (41)	44–100	72.5 (59)	0–100
Extension	78 (46)	1–100	53.5 (57.5)	0–100
Rotation right	79 (57)	19–100	72.5 (50.25)	0–100
Rotation left	71 (66)	18–100	73.5 (45.25)	0–100
Lateral bending right	75 (19)	17–100	75 (50.75)	2–100
Lateral bending left	64 (37)	15–100	71 (51.75)	0–100

Abbreviations: IQR, interquartile range.

**Table 5:** Results of standardized questionnaires on quality of life, impairment, kinesiophobia, and pain in amateur sports after spine trauma at the time of the interview (n = 80).

	SF-36 PCS	ODI [%]	NDI [%]	TSK-17	HADS	Pain 5-Day Maximum *
mean	44.5	19.6	18.0	36.9	11.7	31.2
95% CI	[41.7;47.1]	[15.2;24.0]	[9.4;26.6]	[35.1;38.8]	[9.9;13.6]	[24.1;38.3]
median	46	12	18	37	9	16,5
min	16	0	0	21	0	0
max	68	68	66	60	34	94
IQR	20.25	24	30	13	12.25	59
SD	12.6	18.1	19.1	8.3	8.3	31.9

* Measured on an analog scale from 0 to 100. Abbreviations: SF-36 PCS, Short-Form-36 Physical Component Score; IQR, interquartile range; ODI, Oswestry Disability Index; NDI, Neck Disability Index; TSK-17, Tampa Scale of Kinesiophobia 17 Item Version; HADS, Hospital Anxiety and Depression Scale; CI, confidence interval; IQR, interquartile range; SD, standard deviation.

**Table 6:** Bivariate analyses of differences in patient-reported outcomes concerning quality of life and impairment after spine trauma among subgroups divided by potential influencing factors. The Wilcoxon Signed-Rank Test was used.

	SF-36 PCS	*p*-Value	ODI	*p*-Value	NDI	*p*-Value
	Median (IQR)		Median (IQR)		Median (IQR)	
Time since injury above average (yes/no)	47.0 (16.25)/44.0 (22.25)	0.934	12.0 (27.0)/11.0 (22.5)	0.219	8.0 (20.0)/20.0 (30.0)	0.666
Sport activity						
Returned to sport at time of interview (yes/no)	48.0 (18.75)/41.5 (24.5)	**0.044**	12.0 (23.0)/14.0 (34.0)	0.082	8.0 (25.5)/32.0 (0.0)	0.315
Subjective same level if active in sport (yes/no)	55.5 (9.75)/38.5 (12.0)	**<0.001**	8.0 (9.5)/26.0 (27.25)	**0.001**	3.0 (8.0)/28.0 (26.0)	**0.002**
Patient characteristics						
Age (under mean/over mean)	51.13.75/40.0 (23.5)	**0.005**	8.0 (6.0)/20.0 (28.0)	**0.036**	8.0 (18.0)/14.0 (30.0)	0.545
Sex (female/male)	42.0 (20.5)/49.5 (17.0)	0.342	18.0 (28.0)/11.0 (19.5)	0.141	22.0 (11.0)/8.0 (30.0)	0.716
Charlson Comorbidity Index (0/>0)	48.5 (17.75)/37.5 (15.25)	**0.016**	12.0 (19.5)/36.0 (38.0)	0.153	8.0 (31.0)/20.0 (0.0)	0.761
Preexisting spine pathology (no/yes)	49.5 (16.75)/39.5 (24.50)	**0.007**	9.0 (16.5)/24.0 (29.0)	**0.001**	7.0 (23.0)/20.0 (30.0)	0.268
BMI (≤25/>25)	48.0 (17.0)/44.0 (22.0)	0.245	12.0 (30.0)/12.0 (25.0)	0.777	13.0 (25.5)/8.0 (24.0)	0.640
Injury and therapy						
Only cervical spine affected (yes/no)	53.5 (24.5)/45.0 (18.5)	0.526	n.a.		7.0 (21.5)/24.0 (18.0)	0.186
Multiple regions of the spine affected (no/yes)	48.5 (19.25)/40.5 (9.5)	0.053	11.0 (19.0)/32.0 (24.0)	0.131	8.0 (21.0)/28.0 (11.0)	0.144
Treatment (conservative/surgical)	50.0 (13.0)/44.0 (23.5)	0.081	10.0 (9.0)/20.0 (30.5)	**0.036**	8.0 (16.0)/14.0 (30.5)	0.867
Injury type B, C, or A4 (AO Spine) (no/yes)	48.0 (17.75)/43.0 (24.5)	0.409	10.0 (29.0)/20.0 (24.0)	0.228	14.0 (22.5)/8.0 (27.0)	0.617
Sport injury (no/yes)	45.0 (20)/48.0 (18)	0.345	12.0 (26.0)/8.0 (28.5)	0.482	21.0 (24.5)/0.0 (6.0)	**0.045**
Posttreatment						
Physical therapy (no/yes)	54.0 (12.0)/44.0 (22.5)	0.058	7.0 (8.0)/14.0 (30.0)	**0.019**	0.0 (11.0)/14.0 (25.5)	0.241
Rehabilitation (no/yes)	53.0 (13.25)/37.5 (20.0)	**<0.001**	8.0 (8.5)/24.0 (29.5)	**0.001**	8.0 (20.0)/31.0 (12.0)	**0.023**
Motivation						
At least 5 h sport/week before injury (yes/no)	45.0 (19.0)/48.0 (22.5)	0.843	14.0 (26.0)/11.0 (29.0)	0.63	16.0 (27.5)/8.0 (20.0)	0.564
At least 3 x sport/week before injury (yes/no)	44.0 (19.0)/50.0 (24.5)	0.823	18.0 (20.0)/10.0 (33.5)	0.321	14.0 (25.5)/8.0 (21.0)	0.520

Abbreviations: SF-36 PCS, Short-Form-36 Physical Component Score; IQR, interquartile range; ODI, Oswestry Disability Index; NDI, Neck Disability Index. Bold indicates *p*-Values below 0.05.

**Table 7:** Bivariate analyses of differences in patient-reported outcomes concerning kinesiophobia, depressive symptoms, and pain after spine trauma among subgroups divided by potential influencing factors. Wilcoxon Signed-Rank Test was used.

	Tampa Scale of Kinesiophobia		HADS		Pain 5-Day Maximum	
	Median (IQR)	*p*-Value	Median (IQR)	*p*-Value	Median (IQR)	*p*-Value
Time since injury above average (yes/no)	38.0 (12.0)/34.5 (13.5)	0.728	9.0 (12.75)/8.5 (10.75)	0.861	16.5 (55.5)/14.5 (49.75)	0.234
Sport activity						
Returned to sport at time of interview (yes/no)	35.0 (11.0)/42.0 (13.5)	**0.016**	8.0 (11.75)/14.0 (11.25)	0.050	10.5 (49.75)/34.0 (66.0)	**0.025**
Subjective same level if active in sport (yes/no)	31.0 (9.0)/39.0 (10.0)	**0.002**	6.0 (5.75)/15.5 (14.25)	**<0.001**	4.5 (16.5)/51.5 (66.0)	**<0.001**
Patient characteristics						
Age (under mean/over mean)	34.0 (9.0)/38.5 (14.25)	0.174	7.0 (9.75)/12.0 (12.0)	0.145	12.0 (41.25)/20.0 (66.25)	0.269
Sex (female/male)	38.0 (13.0)/34.0 (13.0)	0.720	11.0 (10.75)/8.0 (12.75)	0.502	47.5 (63.5)/12.0 (42.75)	0.099
Charlson Comorbidity Index (0/>0)	36.0 (13.0)/38.0 (14.5)	0.236	8.0 (12.0)/14.5 (12.75)	0.085	12.0 (48.0)/63.0 (58.25)	**0.015**
Preexisting spine pathology (no/yes)	34.0 (11.25)/39.0 (13.0)	**0.037**	7.5 (12.5)/12.0 (11.0)	0.365	12.0 (44.0)/32.5 (69.0)	0.059
BMI (≤25/>25)	37.0 (10.75)/36.0 (12.0)	0.753	9.0 (10.5)/9.0 (13.0)	0.938	10.0 (61.0)/26.0 (46.0)	0.25
Injury and therapy						
Only cervical spine affected (yes/no)	31.0 (13.0)/37.0 (11.75)	0.701	5.5 (4.0)/11.0 (12.0)	**0.017**	6.5 (16.5)/19.5 (56.75)	**0.033**
Multiple regions of the spine affected (no/yes)	34.0 (13.0)/39.0 (5.5)	0.075	8.0 (11.0)/18.0 (12.25)	**0.018**	13.5 (52.5)/49.5 (50.75)	0.054
Treatment (conservative/surgical)	32.0 (9.0)/38.0 (12.75)	0.058	6.0 (10.0)/10.0 (11.5)	0.164	7. 0 (42.0)/20.0 (62.5)	0.061
Injury type B, C or A4 (AO Spine) (no/yes)	34.5 (13.25)/39.0 (10.5)	0.232	8.0 (12.0)/9.0 (11.25)	0.602	13.5 (56.25)/24.5 (55.75)	0.485
Sport injury (no/yes)	38.5 (12.0)/30.0 (8.0)	**0.005**	9.0 (12.0)/7.0 (11.0)	0.272	17.0 (52.0)/10.0 (62.0)	0.195
Posttreatment						
Physical therapy (no/yes)	32.0 (9.0)/37.0 (12.0)	0.102	8.0 (8.0)/9.0 (12.5)	0.215	2.0 (10.0)/20.0 (60.0)	**0.005**
Rehabilitation (no/yes)	32.0 (9.5)/39.0 (12.25)	**0.002**	6.5 (10.25)/14.0 (15.25)	**<0.001**	6.0 (15.75)/51.5 (64.0)	**<0.001**
Motivation						
At least 5 h sport/week before injury (yes/no)	36.0 (10.5)/37.5 (14.25)	0.748	11.0 (11.25)/8.0 (10.5)	0.150	17.0 (56.25)/15.5 (59.0)	0.797
At least 3 x sport/week before injury (yes/no)	37.0 (10.5)/36.0 (16.0)	0.969	12.0 (11.0)/7.0 (11.5)	**0.031**	19.0 (55.0)/12.0 (69.5)	0.996

Abbreviations: IQR, interquartile range; HADS, Hospital Anxiety and Depression Scale. Bold indicates *p*-Values below 0.05.

## Data Availability

The original contributions presented in the study are included in the article/Supplementary Materials. Further inquiries can be directed to the corresponding author/s.

## Supplementary Materials

The following supporting information can be downloaded at: https://www.mdpi.com/article/10.3390/sports12080213/s1, Structured telephone interview on the resumption of sports activity after spinal injury.

## Abbreviations

CCI	Charlson Comorbidity Index
HADS	Hospital Anxiety and Depression Scale
IQR	interquartile range
NDI	Neck Disability Index
ODI	Oswestry Disability Index
PRO	patient-reported outcomes
PROM	patient-reported outcome measure
ROM	range of movement
RTS	return to sport
SD	standard deviation
SF-36 PCS	Short-Form-36 Physical Component Score
sPILP	subjective preinjury level of performance
TSK-17	Tampa Scale of Kinesiophobia 17 Items
