# Supplementary material for: The Impact of Spine Injuries on Amateur Athletes: An Exploratory Analysis of Sport-Related Patient-Reported Outcomes
Source: Sports (Basel). 2024 Aug 1;12(8):213. doi: 10.3390/sports12080213 (PMC11359861; doi:10.3390/sports12080213)
Supplement: Supplementary file 1 [file sports-12-00213-s001.zip › sports-3088647-supplementary.pdf]

Structured telephone interview on the resumption of sports activity after spinal injury

Participant ID: ____

Sex:
- ☐ male
- ☐ female

Date: _______________

Interviewer (surname, first name):

____

A. General questions about spinal injuries and therapy as well as pre-existing conditions

1. Follow-up treatment for cervical spine injury

1.1 Have you had a cervical spine injury?
- ☐ Yes
- ☐ No

If "No" is ticked, skip the following questions and proceed to "2. Rehabilitation and physiotherapy".

1.2 Did you wear a neck brace after your injury or surgery?
- ☐ Yes
- ☐ No

If "No" is ticked, skip the following questions and proceed to "2. Rehabilitation and physiotherapy".

1.3 Have you worn a soft neck brace or a hard neck brace?
- ☐ Soft neck brace (e.g. made of foam)
- ☐ Hard neck brace (e.g. made of plastic)
- ☐ First a hard, then a soft neck brace

1.4 How long after injury or surgery did you wear a neck brace?
- ☐ 1 week or less
- ☐ 2 weeks
- ☐ 3 weeks
- ☐ 4 weeks
- ☐ 5 weeks
- ☐ 6 weeks
- ☐ Longer than 6 weeks

2. Rehabilitation and physiotherapy

2.1 Did you go to rehab for your spinal injury?
- ☐ Yes

☐ No

If "No" is ticked, skip question 2.2 and proceed to question 2.3 in this section

2.2 How long did your rehab take?

☐ 1 week
☐ 2 weeks
☐ 3 weeks
☐ 4 weeks
☐ Longer than 4 weeks

2.3 Did you have outpatient physiotherapy for your spinal injury?

☐ Yes
☐ No

If "No" is checked, skip the following question and proceed to "3. Operations that have taken place".

2.4 For how long immediately after your injury or operation did outpatient physiotherapy take place without longer interruptions?

☐ Up to 2 weeks
☐ 1 month
☐ 2 months
☐ 3 – 4 months
☐ 5 – 6 months
☐ 7 – 12 months
☐ Longer than 12 months
☐ Because of my spinal injury, physiotherapy still takes place regularly without any longer interruptions.

3. Operations that have taken place

3.1 Have you had surgery for your spinal injury or consequential problems with your spine?

☐ Yes
☐ No

If "No" is ticked, skip the following questions and proceed to "4. Pain and pain medication".

3.2 Has surgery (or multiple surgeries) been performed at another clinic for your spinal injury?

☐ Yes
☐ No

If "No" is ticked, skip the following questions and proceed to "4. Pain and pain medication".

3.3 How many surgeries for your spinal injury have been performed at other clinics in total?

☐ 1
☐ 2
☐ 3
☐ more than 3

3.4 Which of the following types of surgery has been performed at other clinics?

If applicable, multiple answers can be selected.

☐ Removal of the inserted metal
☐ Corrective surgery for a misalignment of the vertebral bodies
☐ Artificial replacement of a vertebral body
☐ Artificial replacement of an intervertebral disc

- ☐ Widening of the narrowed spinal canal
- ☐ Other Operation:

3.5 For what reason did the surgeries performed at other clinics become necessary?
If applicable, multiple answers can be selected.
- ☐ Ache
- ☐ Deformity of the spine
- ☐ Restriction of movement
- ☐ Loosening of the metal or change of position of the metal
- ☐ Infection of the metal or surrounding soft tissues
- ☐ Pressure on the spinal cord or nerve roots with failure/paralysis/numbness
- ☐ Other reasons:

4. Pain and pain medication

4.1 Do you take pain medication regularly (i.e. at least once a month) because of spinal pain?
- ☐ Yes
- ☐ No

If "No" is ticked, skip the following questions and proceed to "5. Pre- and concomitant diseases".

4.2 What kind of painkillers do you take regularly (at least once a month)?
If applicable, multiple answers can be selected.
- ☐ Ibuprofen, diclofenac (Voltaren) and/or aspirin
- ☐ Paracetamol
- ☐ Novaminsulfon (Novalgin)
- ☐ Tramadol (Tramal), Tilidin
- ☐ Oxycodon
- ☐ Hydromorphon (Palladon)
- ☐ Fentanyl
- ☐ Other pain medications:

5. Pre-existing and concomitant diseases

5.1 In addition to the spinal injury that has occurred, are there any other pathological or degenerative changes in your spine?
- ☐ Yes
- ☐ No

If "No" is ticked, skip the following questions and proceed to question 5.3 (height and weight) in this section.

5.2 What other spinal disorders or problems do you know of?
If applicable, multiple answers can be selected.
- ☐ Osteoporosis
- ☐ Herniated disc or disc protrusion
- ☐ Chronic neck or back pain even before the injury
- ☐ Spinal stenosis/narrowing of the spinal canal
- ☐ "Arthrosis" of the spine
- ☐ Spondylolisthesis
- ☐ Skoliose
- ☐ Ankylosing spondylitis (ossifying and stiffening spinal disease)

☐ Other spinal disorders:

5.3 How tall and how heavy are you at the moment?

Size: ___ ___ ___ cm

Weight: ___ ___ ___ kg

5.4 Which of the following other diseases may you be aware of?
If applicable, multiple answers can be selected. If there is no known disease, tick "none".

☐ None
☐ Hypertension
☐ Diabetes mellitus (diabetes)
☐ Circulatory disorders of the heart (angina pectoris)
☐ Heart attack
☐ Cardiac insufficiency
☐ Stroke
☐ Diseases of the kidneys, renal insufficiency
☐ Cancers
☐ Thyroid disorders
☐ Osteoarthritis or inflammation of other joints (not the spine)
☐ Chronic infectious disease
☐ Stomach ulcer
☐ Inflammatory bowel disease
☐ Liver
☐ Asthma bronchiale
☐ Chronic Bronchitis/Chronic Obstructive Pulmonary Disease (COPD)
☐ Blutarmut (Anämie)
☐ Chronic skin diseases (e.g. psoriasis, psoriasis, neurodermatitis)
☐ Migraine
☐ Epilepsy
☐ Parkinson's disease
☐ Depressions
☐ Insomnia

☐ Other diseases: ____

B. Questions about sports activity before and after spinal injury

1. Physical activity before your spinal injury

1.1 Did you exercise regularly before your spinal injury?

☐ Yes
☐ No

If "No" is ticked, skip the following questions and finish section B.

1.2 Did you sustain your spinal injury in a sports accident?

☐ Yes
☐ No

1.3 For example, how many times a week did you exercise before your spinal injury? On average...

☐ less than 1 time a week
☐ 1 to 2 times a week
☐ 3 to 4 times a week
☐ 5 to 6 times a week
☐ daily

1.4 How many hours did you exercise per week before your spinal injury?

- ☐ 30 min to 1 hour
- ☐ 1 – 2 hours
- ☐ 3 – 4 hours
- ☐ 5 hours or more

1.5 What sports did you do the most before your spinal injury?
Please tick only one sport here.

- ☐ Aerobics
- ☐ American Football/Rugby
- ☐ Basketball
- ☐ Bodybuilding/Kraftsport
- ☐ Ice skating
- ☐ Soccer
- ☐ Handball
- ☐ Hockey/Ice Hockey
- ☐ Jog
- ☐ Martial arts
- ☐ Climb
- ☐ Athletics
- ☐ Cycling
- ☐ Ride
- ☐ Row
- ☐ Swim
- ☐ Skiing/snowboarding
- ☐ Dance
- ☐ Tennis
- ☐ Table tennis
- ☐ Gymnastics
- ☐ Volleyball
- ☐ Walking/Wandern
- ☐ Yoga

☐ Other Sportart: ____

1.6 What other sports did you do before your spinal injury?
Multiple selections possible if applicable.

- ☐ none
- ☐ Aerobics
- ☐ American Football/Rugby
- ☐ Basketball
- ☐ Bodybuilding/Kraftsport
- ☐ Ice skating
- ☐ Soccer
- ☐ Handball
- ☐ Hockey/Ice Hockey
- ☐ Jog
- ☐ Martial arts
- ☐ Climb
- ☐ Athletics
- ☐ Cycling
- ☐ Ride
- ☐ Row
- ☐ Swim
- ☐ Skiing/snowboarding
- ☐ Dance
- ☐ Tennis
- ☐ Table tennis
- ☐ Gymnastics
- ☐ Volleyball
- ☐ Walking/Wandern
- ☐ Yoga

☐ Other Sportart: ____

2. Sports activity after spinal injury

2.1 Have you started exercising again after your spinal injury? If you were not active in sports before, do you now do sports after your spinal injury?
Also choose "Yes" if the test person became temporarily active again after the spinal injury, but the sporting activity was discontinued again in the further course.

☐ Yes, I became active (again) after my spinal injury
☐ No, after my spinal injury, I didn't (re)do sports

If "Yes" was ticked, i.e. the test person became (again) active in sports after the spinal injury, skip question 2.2 and continue with question 2.3.

If "No" is ticked, answer question 2.2 and complete the remaining questions in the sections "2. Sports activity after spinal injury" and "3. At the time of resumption of sporting activity".

2.2 After your spinal injury, you did not (anymore) become active in sports. What are the main reasons for this?

☐ I wasn't active in sports even before my spinal injury. (<u>Person does not meet inclusion criteria.)</u>
☐ My spinal injury is the only reason why I didn't do sports anymore.
☐ My spinal injury is not the only, but the decisive reason why I was no longer active in sports.
☐ My spinal injury is one of several equal reasons, but not the only reason why I stopped exercising.
☐ My spinal injury is not the reason why I stopped exercising.

2.3.1 How long after your spinal injury did you start exercising (again)?

☐ Right away
☐ After 1 – 2 weeks
☐ After 3 – 4 weeks
☐ After 5 weeks to 2 months
☐ After 3 – 4 months
☐ After 5 – 6 months
☐ After 7 – 9 months
☐ After 10 – 12 months
☐ After 1 – 2 years
☐ After more than 2 years

If "After 1 – 2 years" or "After more than 2 years" is ticked, skip the following three questions and proceed to 2.4.

2.3.2 How many times a week did you exercise 1 year after your spinal injury? On average

☐ Less than 1 time a week
☐ 1 to 2 times a week
☐ 3 to 4 times a week
☐ 5 to 6 times a week
☐ Daily

2.3.3 How many hours a week did you exercise 1 year after your spinal injury?

☐ 30 min to 1 hour
☐ 1 to 2 hours
☐ 3 to 4 hours
☐ 5 hours or more

2.3.4 Were you able to exercise at the same level as before the spinal injury 1 year after your spinal injury?

☐ Yes
☐ No

2.4 For example, how many times a week do you currently exercise? On average

☐ Less than 1 time a week

- ☐ 1 to 2 times a week
- ☐ 3 to 4 times a week
- ☐ 5 to 6 times a week
- ☐ daily
- ☐ In the meantime, I have stopped my sporting activity again

If the sporting activity has been discontinued in the meantime, answer question 2.5, otherwise skip question 2.5 and continue with question 2.6.

2.5 In the meantime, you have stopped your sporting activity. What are the main reasons for this?

- ☐ My spinal injury is the only reason why I had to stop my sports activity again.
- ☐ My spinal injury is not the only, but the decisive reason why I had to stop my sporting activity again.
- ☐ My spinal injury is one of several equal reasons, but not the only reason why I had to stop my sports activity again.
- ☐ My spinal injury is not the reason why I had to stop my sports activity again.

2.6 How many hours do you currently exercise per week?

- ☐ 30 min to 1 hour
- ☐ 1 to 2 hours
- ☐ 3 to 4 hours
- ☐ 5 hours or more

2.7 Which sports do you currently do most often?

Single Selection

- ☐ Aerobics
- ☐ American Football/Rugby
- ☐ Basketball
- ☐ Bodybuilding/Kraftsport
- ☐ Ice skating
- ☐ Soccer
- ☐ Handball
- ☐ Hockey/Ice Hockey
- ☐ Jog
- ☐ Martial arts
- ☐ Climb
- ☐ Athletics
- ☐ Cycling
- ☐ Ride
- ☐ Row
- ☐ Swim
- ☐ Skiing/snowboarding
- ☐ Dance
- ☐ Tennis
- ☐ Table tennis
- ☐ Gymnastics
- ☐ Volleyball
- ☐ Walking/Wandern
- ☐ Yoga

- ☐ Other Sportart: ____

2.8 What other sports are you currently doing?

Multiple selections possible if applicable.

- ☐ none
- ☐ Aerobics
- ☐ American Football/Rugby
- ☐ Basketball
- ☐ Bodybuilding/Kraftsport
- ☐ Ice skating
- ☐ Soccer
- ☐ Handball
- ☐ Cycling
- ☐ Ride
- ☐ Row
- ☐ Swim
- ☐ Skiing/snowboarding
- ☐ Dance
- ☐ Tennis

- ☐ Hockey/Ice Hockey
- ☐ Jog
- ☐ Martial arts
- ☐ Climb
- ☐ Athletics
- ☐ Table tennis
- ☐ Gymnastics
- ☐ Volleyball
- ☐ Walking/Wandern
- ☐ Yoga

☐ Other Sportart: ______________________________________

2.9 Are you currently able to exercise at the same level as before the spinal injury?

- ☐ Yes
- ☐ No

3. Discomfort at the time of resumption of sports activity

3.1 When you resumed exercise, were you pain-free in the injured section of your spine?

- ☐ Yes, I was pain-free
- ☐ No, I was still in pain

3.2 When you resumed physical activity, was your perceived mobility of the affected section of the spine fully restored?

- ☐ Yes, the mobility felt free
- ☐ No, the mobility was still limited

3.3 When you resumed exercising, did you still have complaints in your arms and legs from the nerve pathways, such as pain radiating from your back to your arms or legs, electrifying pain, muscle weakness of the arms or legs, or numbness/tingling?

- ☐ Yes, such complaints still existed
- ☐ No, there were no such complaints (anymore)

3.4 Which of the following problems do you continue to have when exercising?
Multiple selections possible, if applicable. If there are no problems, tick "none".

- ☐ None
- ☐ Vertigo
- ☐ Neck pain or back pain
- ☐ Radiating pain, electrifying pain in arms or legs
- ☐ Muscle weakness in the arms or legs
- ☐ Numbness or tingling in the arms or legs
- ☐ Fear of re-injury to the spine

☐ Other problems: ____

End telephone interview.

Notes:
